# The spectrum of primary immunodeficiencies at a tertiary care hospital in Pakistan

**DOI:** 10.1016/j.waojou.2020.100133

**Published:** 2020-08-05

**Authors:** Sonia Qureshi, Fatima Mir, Samina Junejo, Khalid Saleem, Samreen Zaidi, Abdullah B. Naveed, Khalil Ahmad, Farah Naz Qamar

**Affiliations:** a Department of Pediatrics and Child Health, Aga Khan University, Stadium Road, Karachi, 74800, Pakistan; b Department of Pediatrics, The Indus Hospital, Korangi Road, Karachi, Pakistan; c Children's Hospital and The Institute of Child Health, Multan, Pakistan; d National Institute of Blood Disease & Bone Marrow Transplantation, P.E.C.H.S, Karachi, Pakistan; e Medical College, Aga Khan University, Stadium Road, Karachi, 74800, Pakistan

**Keywords:** Children, Primary immunodeficiency disorders, Chronic granulomatous disease, Consanguineous marriages, PIDs, Primary Immunodeficiency Disorders, NGS, Next-Generation Sequencing, WES, Whole Exome Sequencing, NBT, Nitrotetrazolium blue test, DHR, Dihydrorhodamine, CGD, Chronic Granulomatous Disease, SCID, Severe Combined Immunodeficiency Disorder, LAD, Leukocyte Adhesion Deficiency, HPS, Hermansky-Pudlak Syndrome, ICF-2, Immunodeficiency Centromeric Instability and Facial Anomalies Syndrome, TRES, Trichohepatoenteric syndrome, LMIC, Low Middle Income Countries, USA, United States of America, I/V, Intravenous, S/C, Subcutaneous, ARDS, Acute Respiratory Distress Syndrome, BCG, Bacille Calmette-Guerin, OPV, Oral Polio Vaccine, VDP, Vaccine Derived Poliovirus, BMT, Bone Marrow Transplant, AFIP, Armed Forces Institute of Pathology

## Abstract

**Background:**

Primary Immunodeficiency Disorders (PIDs) are well-known disorders in the West. but the recognition and diagnosis of these disorders is challenging in developing countries. We present the spectrum of PIDs seen at a tertiary care center in Pakistan, identified using clinical case definitions and molecular methods.

**Methods:**

A retrospective chart review of children suspected to have PID was conducted at the Aga Khan University Hospital (AKUH) Karachi, Pakistan from 2010 to 2016. Data on demographics, clinical features, family history of consanguinity, sibling death, details of laboratory workup done for PID and molecular tests targeted panel next generation sequencing (NGS) or whole exome sequencing (WES) performed at the Geha laboratory at Boston Children’s Hospital, USA was collected. The study was exempted from the Ethical Review Committee of AKUH.

**Results:**

A total of 43 children visited the hospital with suspected PID during the study period. Genetic testing was performed in 31/43 (72.1%) children. A confirmed diagnosis of PID was established in 20/43 (46.5%) children. A pathogenic gene variant was identified in 17(85%) of the 20 confirmed cases (Table 1). Twelve (60%) of the confirmed cases of PID were male. The most common presenting symptom was recurrent diarrhea 11/20 (55%). The mean (±S.D) age of the cases at the time of diagnosis was 4.2 (±4.1) years. Chronic granulomatous disease (CGD) was the most common 6/20 (30%) disorder, followed by severe combined immunodeficiency (SCID) 3/20 (15%), leukocyte adhesion deficiency (LAD) 3/20 (15%), agammaglobulinemia/hypogammaglobulinemia 3/20 (15%), and Hermansky-Pudlak Syndrome (HPS) 2/20 (10%). Wiskott-Aldrich Syndrome, Immunodeficiency Centromeric Instability and Facial Anomalies Syndrome (ICF 2), Trichohepatoenteric syndrome (TRES), and C3 deficiency were each diagnosed once {1/20 (4.3%) each} (Table 1). Of these 20 confirmed cases, almost all 19/20 (95%) had a family history of consanguinity. Sibling death was reported in 5/20 (25%) of these cases. Five out of the 20 (25%) children died over the 7-year period for various reasons.

**Conclusion:**

PIDs are not uncommon in Pakistan; their diagnosis may be missed or delayed due to the overlapping of clinical features of PID with other diseases and a lack of diagnostic facilities. There is a need to build capacity for early recognition and diagnosis of PIDs to decrease morbidity and mortality.

## Introduction

Primary immunodeficiency disorders (PIDs) are a heterogeneous group of genetic disorders characterized by an impaired ability of the immune system to produce a normal immune response. This is due to inherited defects in either cellular or humoral immunity, which results in a spectrum of issues such as recurrent infections, allergies, autoimmunity, and malignancies.^1^^,^^2^ In neonates, PIDs often present with severe infections leading to death; whereas in adolescents these infections are less severe albeit recurrent. Diagnosing PIDs is challenging because of the variability in clinical presentation and limited availability of diagnostic tests, particularly in low middle-income countries (LMIC). When diagnostic tests are available, their cost often becomes a limiting factor. Advances in molecular diagnostic techniques and the identification of known gene defects have helped to facilitate the diagnosis of patients with PIDs.^3^ The true global prevalence and distribution of PIDs remain unclear. The prevalence statistics available from nationwide registries are mostly derived from limited areas of the world. The data obtained from these registries often underestimate the true prevalence, because not all cases are reported to these registries, and due to ambiguity in what constitutes a PID case, some cases are missed. These issues are compounded in developing countries because of the lack of physician training in identification of these disorders and the limited access to diagnostics in these countries. Recent studies have shown that PIDs are more common than previously thought, and that around 1% of the population may have an underlying PID.^4^ The burden of PID varies by region, being highest in the United States of America (USA), followed by Europe, Latin America, Middle East, Asia, and finally Africa.^4^ This frequency may be biased by the availability of resources for diagnosis of these disorders.

Most PIDs are autosomal recessive, which makes it safe to assume that the incidence of PIDs is greater in regions having higher rates of consanguinity. However, limited studies have been carried out in such regions and hence the burden and type of PIDs in such areas is relatively unknown. Consanguineous marriages are common in developing Asian countries ranging between 20 and 70% of all marriages, and Pakistan is no different, with about 70% of the marriages being consanguineous.^5^, ^6^, ^7^ The data on PIDs in Pakistan is currently confined to a few case reports, case series, and editorials; there are no detailed reports on the spectrum of PIDs seen in the country.^8^, ^9^, ^10^, ^11^

Diagnosing and treating PIDs is a challenge in Pakistan. Patients often die before the disorder is recognized by a physician, leading to a delay in diagnosis or death of the child. Families are often referred to tertiary care centers following the death of a sibling or for admission for life-threatening infections. Even in large tertiary care centers with well-equipped laboratories, there are limited diagnostic facilities available for confirming PIDs. Even when these are available, the diagnostic tests are expensive and beyond the reach of most families as the health care system of Pakistan relies on out-of-pocket payments for health expenditures. Once the diagnosis of PID is confirmed, supportive therapies like antibiotic and antifungal prophylaxis, intravenous (I/V), or subcutaneous (S/C) immunoglobulin therapy, depending on the underlying disorder, are offered. Intravenous immunoglobulin (IVIG) is the standard therapy for most humoral deficiencies, but it is expensive and beyond the reach of most patients in low middle income countries (LMICs). S/C immunoglobulins provide ease of administration, but their availability is limited in LMICs. Bone marrow transplant is available in certain settings but has its limitations in countries like Pakistan where no donor registries are available, and the cost of the transplant coupled with the risk of infections in the post-transplant period make this option challenging to pursue. Gene therapy is another promising albeit costly treatment option undergoing experimentation and is now considered an option for the treatment of multiple non-life-threatening disorders (ie, immunological disorders and systemic protein deficiency). This study aims to report the spectrum of PID cases observed at a tertiary care center in Pakistan, with a focus on the molecular diagnosis in this patient population over the last 7 years.

## Materials and methods

The Aga Khan University Hospital (AKUH) is a 700 bed, not-for-profit tertiary care hospital in Karachi, Pakistan. The hospital receives referrals from all over the country. A retrospective chart review of children suspected to have PID was conducted at the AKUH, Pakistan from 2010 to 2016. Data on demographics, clinical features, age at onset of symptoms, age at presentation, history of recurrent infections, skin allergies, family history of consanguinity, sibling death, details related to laboratory workup done for PID (complete blood count, serum immunoglobulin levels, nitrotetrazolium blue test (NBT), dihydrorhodamine(DHR) and flow cytometric analysis) were collected. Molecular tests targeted panel next generation sequencing (NGS) or whole exome sequencing (WES) were performed at Geha laboratory at Boston Children’s Hospital, USA. Targeted panel NGS was performed using the PID v2 panel and Ion Torrent™ S5 sequencer (ThermoFisher), with an average coverage of 328x. Variant annotation was performed with VarSeq™ software (Golden Helix). Whole exome sequencing (WES) was performed using a previously described pipeline with an average on-target coverage of 80×.^12^ The cost of shipping and molecular testing was covered by a philanthropic grant from the Perkins Fund. Data were analyzed using IBM SPSS Statistics v. 20.0. Mean ± S.D is reported for quantitative variable like age at diagnosis, delay in diagnosis (days), etc. Frequency and percentages are reported for categorical variables such as gender, type of PID, clinical features, history of sibling death, consanguinity in family, mortality, etc.

## Results

**Fig. 1:**
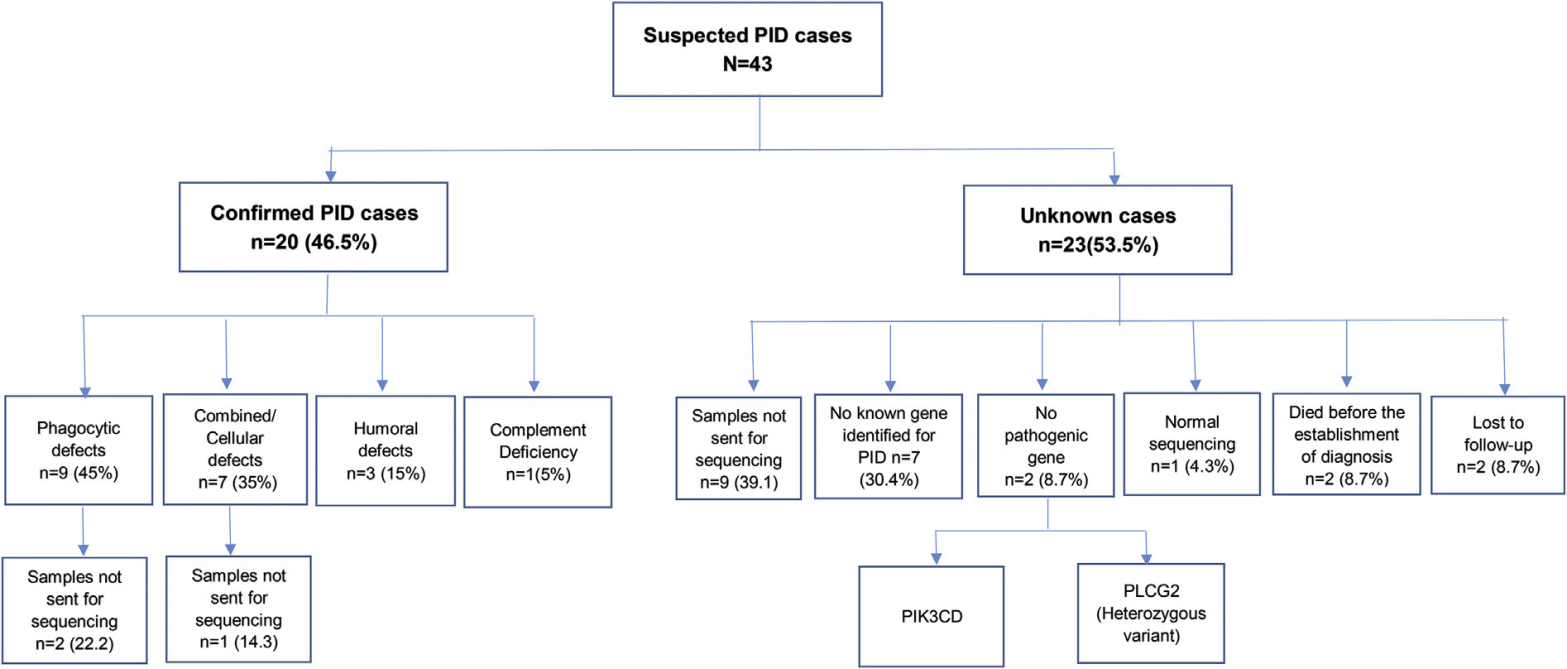
Study flow showing distribution of study subjects

A total of 43 children visited the hospital with a suspected PID during the study period. Among them, genetic testing was performed in 31/43 (72.1%) children. A confirmed diagnosis was established in 20/43 (46.5%) children. A pathogenic gene variant was identified in 17 (85%) of the 20 confirmed cases (Table 1). Of the remaining 23 patients, samples for 9 patients were not sent for molecular testing, 7 had no known genetic mutation for PID (Table 2), no pathogenic genetic variant was identified in 2 patients, 2 died before a final diagnosis could be established, 2 were lost to follow-up, and 1 had normal sequencing results (Fig. 1). Twelve (60%) of the 20 confirmed children with PID were male. The mean (±S.D) age at the time of diagnosis was 4.2 (±4.1) years. The mean (±S.D) delay in diagnosis was 1333 ± 1420 days. The most common presenting symptom was recurrent diarrhea 11/20 (55%) followed by recurrent pneumonia 8/20 (40%), oral thrush 7/20 (35%), skin rashes and abscesses 6/20 (30%), osteomyelitis 1/20 (5%), and meningitis 1/20 (5%). Chronic granulomatous disease (CGD) was the most common 6/20 (30%) disorder, followed by severe combined immunodeficiency (SCID) 3/20 (15%), leukocyte adhesion deficiency (LAD) 3/20 (15%), agammaglobulinemia/hypogammaglobulinemia 3/20 (15%), and Hermansky-Pudlak Syndrome (HPS) 2/20 (10%). Wiskott-Aldrich Syndrome, Immunodeficiency Centromeric Instability and Facial Anomalies Syndrome (ICF 2), Trichohepatoenteric syndrome (TRES), and C3 deficiency were each diagnosed once {1/20 (4.3%) each} (Table 1). Of these 20 confirmed cases, almost all 19/20 (95%) had a family history of consanguinity. Sibling death was reported in 5/20 (25%) of these cases. Five out of 20 (25%) children died due to various reasons. A pathogen (*Pneumocystis jiroveci*) was identified in only 1 of the 5 deceased patients. Among the deceased children, 2 developed acute respiratory distress syndrome (ARDS), 2 died of sepsis, and 1 had plastic bronchitis.

**Table 1:** Clinical and Laboratory characteristics of patients with a confirmed Primary immunodeficiency disorder.

S.No	Age/Sex	Clinical Features	Consanguinity	Sibling Death (Age/Cause of death)	aSpecial Investigations (Normal range)	Mutated Gene	Final Diagnosis	Age at onset of symptom	Age at diagnosis	Outcome
	**Phagocyte Defects (n** = **9)**									
1	11 mo/M	Recurrent abscesses and osteomyelitis	Yes	Yes (18 mo old brother & 16 days old brother with no apparent cause)	**NBT**: Negative **DHR:** Positive **Immunoglobulins** IgG:15.2 (6.5–16) IgA: 3.68 (0.4–3.5) IgM:1.15 (0.5–3.0) IgE: not done **Flow cytometry** Not done **HIV-serology**: not done	**Gene** NCF1 **Loss of coverage** Exon 2 could not be amplified, suggesting a deletion that includes this exon	AR CGD	Birth	6 yr	Alive
2	3.3 yr/M	Recurrent abscesses, lymphad enopathy	No	Yes (9 mo old brother due to pneumonia)	**NBT**: Negative **DHR**: Not done **Immunoglobulins** IgG:23.1 (5.7–17.1) IgA: 3.58 (0.47–2.5) IgM:2.38 (0.64–2.7) IgE: not done **Flow Cytometry** CD3: 5203 (1500–2900) CD4: 2328 (1000–2100) CD8: 1999 (700–1100) CD19: 2407(500–1200) CD 56: 556 (300–600) **HIV-serology**:Negative	**Gene** CYBB **Zygosity** Homozygous **Genomic coordinate** c.1222G > A **Amino acid Change** p.Gly408Arg	X-linked CGD	9 mo	8 yr 9 mo	Alive
3	3 mo/M	Recurrent diarrhea, perianal abscess, skin rash	Yes	No	**NBT**: Negative **DHR**: Not done **Immunoglobulins** IgA: 0.2 (0.09–1.07) Others: not done **Flow Cytometry** CD3: 1652(1800–3300) CD4: 1224 (900–2300) CD8: 237 (700–1500) CD19: 839(700–1700) CD 56: not done **HIV status:** not checked	**Gene** NCF1 **Zygosity** Homozygous **Genomic coordinate** c.294_295insA **Amino acid change** p.Gly99fs	AR CGD	2 mo	2 yr 9 mo	Alive
4	1.3 yr/F	Recurrent diarrhea, cow milk protein allergy	Yes	Yes (1.5 years old sister due to septic shock & had similar complaints, no workup done for PID)	**NBT**: Absent activity **DHR**: Not done **Immunoglobulins** IgG:13.4 (6.5–16.0) IgA:2.49 (0.4–3.5) IgM:1.14 (0.5–3.0) IgE: 1336 (0.4–351.6) **Flow cytometry** CD3: 5030 (1900–2900) CD4: 3617 (1000–1800) CD8: 1388 (700–1200) CD19: 5073(600–1400) CD56:1279 (300–600) **HIV status:** not checked	**Gene** NCF2 **Zygosity** Homozygous **Genomic coordinate** Chr1:183532647 **Loss of coverage**: 1098_1099delTC = p.Gln367fs (Frameshift deletion)	AR CGD	Birth	2 yr	Alive
5	3 yr/M	Recurrent pneumonias, oral thrush, perianal abscess lymphadenopathy	Yes	No	**NBT**: Normal **DHR:** Not done **Immunoglobulins** IgG: 7.62 (.05–14.0) IgA:1.56((0.18–1.7)) IgM: 1.20(0.63–2.9) IgE: >2000(0.4–351.6) **Flow cytometry** CD3: 6124(1500–2900) CD4: 3355(1000–2100) CD8: 2539(700–1100) CD19:1830(500–1200) CD56:not done **HIV serology**: Negative	**Gene** NCF1 **Zygosity** Homozygous **Genomic coordinate** Chr7: 74191664 **Amino acid change** p.Arg42Trp	AR CGD	2 yr	4 yr 5 mo	Alive
6.	7 yr/M	Recurrent pneumonia	Yes	No	**NBT**: Not done **DHR**: Positive I**mmunoglobulins** Not done **Flow cytometry** Not done **HIV status**: not checked	Not done	CGD	9 mo	7 yr	Alive
7.	3.5 mo/F	Delayed shedding of umbilical cord, recurrent oral thrush, skin rash,	Yes	No	**Immunoglobulins** Not done **Flow cytometry** Not done **HIV status**: not checked	**Gene** FERMT3 **Zygosity** Homozygous **Loss of coverage** (Kindlin-3)c.126_126delC = p.Ile42fs (Frameshift deletion)	LAD3	2 mo	1yr 4 mo	Alive
8.	7 mo/F	Delayed shedding of umbilical cord, recurrent diarrhea, skin rash	Yes	No	**Immunoglobulins** IgG:4.02 (6.5–16) IgM:1.36 (0.5–3.0) IgA: 0.36 (0.4–3.5) IgE: 31.0 (1.4–52.3) **Flow Cytometry** CD3: 3987 (2800–3500) CD4: 3122 (1700–2400) CD8: 784 (800–1100) CD 56: 444 j(300–800) CD19: 2168 (1000–1700) **HIV status:** not checked	**Gene** ITGB2 **Zygosity:** Homozygous **Genomic coordinate** (GRCh37/hg19): c.134G > A **Amino acid change** p.Trp45Ter (Nonsense)	LAD1	12 days	5 mo	Alive
9.	4 mo/M	Delayed shedding of umbilical cord and umbilical abscess	Yes	No	c**Flow cytometry for LAD markers:** CD11C deficiency	Not done	LAD2	Birth	3 mo	Alive
	**Combined/Cellular defects (n** = **7)**									
10.	4 mo/M	Recurrent diarrhea, skin rash	Yes	No	**Immunoglobulins** IgG: <0.7 (6.5–16.0) IgA: <0.15 (0.4–3.5) IgM: 0.0 (0.5–3.0) IgE: 440 (1.4–52.3) **Flow Cytometry** CD3: 150 (1800–3300) CD4: 150 (900–2300) CD8: 1 (700–1500) CD56:108 (300–800) CD19: 0 (700–1700) **HIV status:** not checked	**Gene** RAG2 **Zygosity** Homozygous **Genomic coordinate** c.1247G > T **Amino acid change** p.Trp416Leu	SCID Omenn syndrome	1.5 mo	5 mo	Alive
11.	5 mo/F	Recurrent pneumonia, diarrhea, otitis media, skin rash	Yes	No	**Immunoglobulins** IgG:0.66 (1.9–7.9) IgA: <0.15 (0.01–0.59) IgM: not done IgE: 18 (1.4–52.3) **Flow Cytometry** CD3: 428 (2800–3500) CD4: 283 (1700–2400) CD8: 122 (800–1100) CD56: not done CD19: 25(1000–1700) **HIV serology**: Negative	**Gene** RAG1 **Zygosity** Homozygous **Genomic coordinate** Chr 11: 36597688_A > T = c. 2834 A > T **Amino acid change** p.H945L **SIFT** (0.01); **Polyphen** (0.969).	SCID	3 mo	6mo	Died due to bARDS and bPCP pneumonia (1 month *before establishing diagnosis*)
12.	1 yr/F	Recurrent diarrhea, oral thrush	Yes	No	**Immunoglobulins** Ig G: 0.83 (not available) IgA: 0.17 (not available) IgM: 0.23 (not available) IgE: not done **Flow cytometry** CD3: 32 (not available) CD4: 6 (not available) CD8: 23 (not available) CD19: 397 (not available) **HIV serology**: Negative	**Gene** JAK3 **Amino acid change** p.P402L. **SIFT** (0). **Polyphen** (0.85)	SCID	8 mo	1 yr 2 mo	Died due to bARDS (2 months *before establishing diagnosis*)
13	2.5 yr/M	Oral thrush, skin rash, Food allergy, anemia, thrombocytopenia	Yes	No	**Immunoglobulins** Ig G:19.4 (not available) Ig A: 2.99(not available) Ig M: 0.76(not available) Ig E: >5000(not available) **Flow Cytometry** CD3: 1126(not available) CD4: 285(not available) CD8: 675(not available) CD19: 75(not available) CD56: 299(not available) **HIV status**: not checked	**Gene** WAS **Zygosity** Hemizygous **Genomic coordinate** ChrX: 48543941 (Deletion of C)Arg94fs (Frameshift deletion)	WAS	9 mo	3 yr 1 mo	Died due to sepsis (5 months *before establishing diagnosis*)
14.	9 mo/F	Recurrent diarrhea, pneumonia, oral and perianal candidiasis	Yes	No	**Immunoglobulins** IgG: 4.6 (6.5–16) IgA: <0.33 (0.4–3.5) IgM: 0.21 (0.5–3.0) Ig E: 1.23 (1.4–52.3) **Flow cytometry** CD3: 4286 (1900–2900) CD4: 2942 (1000–1800) CD8: 824 (700–1200) CD56: 331 (300–600) CD19: 944 (600–1400) **HIV status:** not checked	**Gene** ZBTB24 **Zygosity** Homozygous **Genomic coordinate** Chr6:109802832 c.396_397delTA = p.His132fs (Frameshift deletion)	ICF 2	4 mo	1 yr 10 mo	Died due to Plastic bronchitis. (2.5 months *after diagnosis*)
15	6 yr/M	Recurrent diarrhea, pneumonia, occulo-cutaneous albinism, blond hairs, easy bruisibility, oral thursh	Yes	No	**Immunoglobulins** Ig G: 9.7 (6.5–16) Ig A: 4.0 (0.4–3.5) IgM: 0.28 (0.5–3.0) IgE: 28 (0.5–393) **Flow cytometry** Not done **HIV status:** not checked	No pathogenic variant found *(Diagnosed on the basis of clinical findings and family history of similar illness in his first cousin)*	Hermansky-Pudlack syndrome	Birth	7 yr	Alive
16.	16 y/F	Recurrent diarrhea, pneumonia, occulo-cutaneous albinism, blond hairs, easy bruisibility, oral thrush, epistaxis, otitis media	Yes	Yes (2.5 years old brother due to pneumonia & had similar features and complaints)	**Immunoglobulins** Ig G:17.8 (6.5–16) Ig A: 4.48 (0.4–3.5) IgM: 0.45 (0.5–3.0) IgE: 12.0 (1.9–170) **Flow cytometry** CD3:1330 (720–2320) CD4: 592 (430–1010) CD8: 687 (170–1050) CD19:175 (100–430) CD56: 48 (100–430) **HIV serology**:Negative	Not done *(Diagnosed on the basis of clinical findings and family history of similar illness in her first cousin)*	Hermansky-Pudlack syndrome	1 yr	17 yr	Alive
	**Humoral/Antibody defects (n** = **3)**									
17.	3 yr/M	Recurrent diarrhea Recurrent abscesses	Yes	No	**Immunoglobulins** IgG: <0.7 (5.7–17.1) IgM: <0.08 (0.64–2.7) IgA: <0.15 (0.47–2.5) IgE: 51 (0.4–351.6) **Flow cytometry** CD3: 1444 (1500–2900) CD4: 389 (1000–2100) CD8: 1064 (700–1100) CD19: 2 (500–1200) CD56: 165 (300–600) **HIV serology**: Negative	**Gene** BTK **Loss of coverage** frameshift deletion resulting in exon18 = c.1883delC = p.T628fs	X-linked Agammaglobulinemia	2yr	4yr 9 mo	Died due to sepsis (4 months *before establishing diagnosis*)
18.	8 yr/F	Recurrent diarrhea and pneumonia	Yes	No	**Immunoglobulins** IgG:1.11(7.3–15.1) IgM: 0.14(0.55–2.1) IgA: <0.15 (0.7–3.25) IgE: <0.1 (0.5–393) **Flow cytometry** CD3: 3472(1300–2200) CD4: 1513 (600–1100) CD8: 1787(500–1000) CD19: 49(300–500) CD56:176(300–500) **HIV status:** not checked	**Gene** TCF3 **Zygosity** Homozygous **Loss of coverage** Total loss of coverage of exons 5–11.	Agammaglobulinemia	1 yr	8 yr 7 mo	Alive
19.	1.9 yr/M	Recurrent pneumonia	Yes	Yes (5 years old brother due to measles)	**Immunoglobulins:** IgG: 9.64 (6.5–16.0) IgM: 0.6 (0.5–3.0) IgA: 0.69 (0.4–3.5) IgE: 3.37 (0.4–351.6) **Flow cytometry:** CD3:1653 (1500–2900) CD4:1242 (1000–2100) CD8: 427 (700–1100) CD19: 12 (500–1200) CD56: 132 (300–600) **HIV status:** not checked	**Gene** TTC37 **Zygosity** Homozygous **Genomic coordinates** (GRCh37/hg19): c.1864A > C **Amino acid change** p.Thr622Pro Polyphen: 0.89 **SIFT**: 0.41	Trichohepatoenteric syndrome (THES)	Birth	1 yr 10 mo	Alive
	**Complement Deficiency (n** = **1)**									
20.	4.5 yr/M	Recurrent meningitis	Yes	No	**Complement Level** C3: 0.05 g/L(0.8–1.5)	**Gene** C3 **Zygosity** Homozygous **Genomic coordinates** (GRCh37/hg19): Chr19: 6711208 C > A (5′ Splicesite, Exon 12)	C3 deficiency	1 yr	5 yr	Alive

a CD count unit: cells/mm^3^,Immunoglobulin unit: G/L, IgE unit: IU/ml, NBT: Nitrotetrazolium blue test, DHR: Dihydrorhodamine.

b ARDS: Acute Respiratory Distress Syndrome, PCP: Pneumocystis carini

c Sample sent to Armed Forces of Institute of Pathology (AFIP), Rawalpindi to check the CD markers for Leukocyte Adhesion Defect (LAD) on flow cytometry

**Table 2:** Clinical and laboratory characteristics of suspected primary immunodeficiency patients with No known gene mutation for PID.

S.No	Age/Sex	Clinical Features	aSpecial Investigations (Normal range)	Suspected diagnosis	Gene Sequence Result	Age at onset of symptom	Age at diagnosis	Outcome
1	10 mo/M	Recurrent diarrhea	**Immunoglobulins** IgG:<0.7 (1.9–7.9) IgA:<0.15 (0.01–0.59) IgM:<0.08 (0.09–2.1) IgE:1.0 (1.4–52.3) **Flow cytometry** CD3:166(1800–3300) CD4:132(900–2300) CD8: 27 (700–1500) CD19: 22(700–1700) CD56: 530(300–800) **HIV serology:** negative	T^−^B^+^NK^−^SCID	No Known candidate gene for PID identified	Birth	10 mo.	Died due to sepsis
2	2.5 yr/M	Work up was done because of family history of SCID.	**Immunoglobulins** Not done **Flow cytometry** CD3:503(1500–2900) CD4: 451(1000–2100) CD8: 68(700–1100) CD19:12(500–1200) CD56: not done **HIV status**: not checked	T^−^B^-^NK^-^ SCID	No Known candidate gene for PID identified	10th day of life	1 yr 8 mo.	Died due to sepsis
3	6 mo/M	Fever, leg gangrene, seizures	**Immunoglobulins** IgG:10.7(6.5–16) IgA: 0.31(0.4–3.5) IgM:0.2(0.5–3) IgE:12.4(1.4–52.3) **Flow Cytometry** CD3:1140(1800–3300) CD4:735(900–2300) CD8: 449(700–1500) CD56: 49 (300–800) CD19: 273(700–1700) **HIV serology:** negative	SCID	No Known candidate gene for PID identified	1.5 mo.	4 mo.	Alive No recurrence of infections in follow up. Less likely to be a PID
4	13 yr/M	Recurrent diarrhea and joint swelling	**Immunoglobulins** IgG: 1.11(5.7–17.1) IgA: 0.41(0.47–2.5) IgM: 0.05(0.64–2.7) IgE: 2.85(1.9–170) **Flow Cytometry** CD3:1718(1100–2200) CD4: 651(600–1600) CD8: 850(500–1200) CD19: 454(200–600) CD56: 83(300–600) **HIV status:**not checked	No	No Known candidate gene for PID identified	6 yr.	13 yr.	Alive
5	8 mo/F	Fever and seizures	**Immunoglobulins** IgG:13.54(6.5–16) IgA: 0.19 (0.4–3.5) IgM: 2.8(0.5–3) IgE: not done **Flow Cytometry** CD3:1624(2800–3500) CD4: 592(1700–2400) CD8: 831(800–1100). CD19: 425(1000–1700) CD56: 913(300–800) **HIV status:** not checked	No	No Known candidate gene for PID identified	4.5 mo.	6 mo.	Alive
6	11 mo/M	Recurrent diarrhea and pneumonia	**Immunoglobulins** IgG:4.96((6.5–16)) IgM:0.09(0.5–3) IgA: 0.16(0.4–3.5) IgE:1.0(1.4–52.3) **Flow Cytometry** CD3: 244(1800–3300) CD4: 105(900–2300) CD8: 146(700–1500) CD19:1.0(700–1700) CD56: 89(300–800) **HIV status**: Negative	SCID	No Known candidate gene for PID identified	4 mo.	11 mo.	Alive No recurrence of infections in follow up. Less likely to be a PID
7	5.5 mo/M	Recurrent pneumonia, skin rash and hyperglycemia	**Immunoglobulins** IgG:0.26(6.5–16) IgA: 0.09(0.4–3.5) IgM:0.12(0.5–3) IgE:5.96 (1.4–52.3) **Flow cytometry** CD3: 2244(1800–3300) CD4: 879 (900–2300) CD8: 1167(700–1500) CD56:196 (300–800) CD19: 21 (700–1700) **HIV status**: not checked	Early onset diabetes and Agammaglobulinemia STAT3 GOF? LRBA?	No Known candidate gene for PID identified	2 mo.	5 mo.	Alive Recurrent otitis media, eczematous skin rash and failure to thrive

a CD count unit: cells/mm^3^,Immunoglobulin unit: G/L, IgE unit: IU/ml

## Discussion

In this case series, 85% of the children were able to get a molecular diagnosis through the support of Boston Children’s Hospital. CGD was the commonest disorder observed. This is consistent with data reported from other Asian countries in which phagocytic defects were the most common type of PIDs ranging from 29% to 60%.^13^, ^14^, ^15^ Almost all cases in this study had a family history of consanguinity. Death of siblings was seen in 25% of the cases. Out of the children who died during the study period, 80% of them were diagnosed after they had died.

Due to a lack of facilities, diagnosing PIDs continues to be a challenge in developing countries. Partnering with international organizations is crucial in aiding and establishing the diagnosis of suspected PIDs in these countries.^9^

Patients with PIDs are often diagnosed based on a clinical history of recurrent infections due to atypical or less virulent pathogens.^16^ However, they can also present with non-infectious manifestations, such as autoimmune disease, or albinism.^17^ Verma et al published a case series of 27 PID cases in whom recurrent pneumonia and recurrent diarrhea were the most common presentations. In our case series, recurrent diarrhea was the most common clinical presentation followed by recurrent pneumonia, oral thrush, skin rash, and abscesses.^18^

Live vaccines (eg, BCG, OPV) should not be administered to children with PIDs due to the risk of developing mycobacterial disease with the bacillus Calmette−Guérin (BCG) vaccine or Vaccine Derived Poliovirus (VDP) with the oral polio virus vaccine (OPV).^19^ Shahmohammadi S et al reviewed 17 cases of disseminated BCG infection in Iran from 2005 to 2010 and observed that 10 (59%) out of the 17 cases had impaired immunity due to an incompetent immune system.^20^ In Pakistan, these vaccines (BCG and OPV) are a part of the national immunization programme; hence, they are administered routinely, much earlier before a diagnosis of immunodeficiency can be suspected or established in children. In our case series, almost all cases were vaccinated with the BCG and OPV at birth; so far, none of them have developed any sign of disease due to the vaccination (VDP or Tuberculosis). Although this report is based on a limited number of cases, it does raise the need for a newborn screening program in the long term, so that children suspected of having a PID do not receive a live vaccine at birth.

Early recognition and prompt diagnosis of PIDs help in preventing significant disease related morbidity and mortality. A clinical history and physical examination together with supportive laboratory investigations can provide clues about an underlying PID. Nevertheless, molecular testing plays an essential role in not only confirming the diagnosis but also in identifying the exact nature of the defect. It also assists in patient care as it allows for prognostic counseling.^21^

Varying degree of support for diagnosing PIDs is available from different institutions and philanthropic foundations such as the (International Union of Immunological Sciences (IUIS), European Society for Immunodeficiencies (ESID), Immune Deficiency Foundation (IDF), and Jeffrey Modell Foundation JMF). In most LMICs, diagnostic and therapeutic services are sparse and/or inaccessible. Armed Forces Institute of Pathology (AFIP) in Rawalpindi, Pakistan is a center where limited immune diagnostics are available but molecular techniques are still lacking. Furthermore, this is the only center where some form of diagnostics is available for PIDs. For a country with a population of 200 million and a birth rate of 29.8 births per 1,000 people, a single facility is not enough to meet the demands.

To our knowledge, this is the first report of PID cases from Pakistan confirmed with molecular diagnosis. The cases reported here only reflect cases referred to a large tertiary care center and represent only the tip of the iceberg.

As a next step, we plan to set up a national registry to bring together individual efforts so that medical care for affected individuals can be improved. Having a national registry will facilitate exchange of experience in the diagnosis and management of PIDs with international registries and will help push policies for support of children with an identified PID. We plan to work towards achieving philanthropic support for IVIG/SCIG or bone marrow transplant (BMT) for the treatment of diagnosed patients. Hopefully, these collective efforts will help with early identification of PIDs, aid in tracking and establishing epidemiological data of PIDs in Pakistan, and help decrease the mortality of patients in developing countries around the world.

## Conclusion

PIDs are not uncommon in Pakistan; their diagnosis may be missed or delayed due to the overlapping of clinical features of PIDs with other diseases and a lack of diagnostic facilities. There is a need to increase testing capacity for early recognition, diagnosis and management of PIDs to decrease morbidity and mortality. There is also a need to establish a national registry for estimating the true burden of PIDs in the country to aid in policy recommendations.

## Funding disclosure

There was no funding for this study.

## Ethical consideration

The study was exempted by Ethical Review Committee (ERC) of Aga Khan University Hospital Karachi (4652-Ped-ERC-17).

## Author's contribution

Sonia Qureshi and Farah Naz Qamar contributed to the study conception and design. Material preparation, data collection and analysis were performed by Sonia Qureshi. Sonia Qureshi, Farah Naz Qamar, Fatima Mir, Samina Junejo, Khalid Saleem, Samreen Zaidi and Khalil Ahmad were involved in patient care and management of PID cases. The first draft of the manuscript was written by Sonia Qureshi and all authors commented on the previous manuscript. Abdullah. B. Naveed contributed in the revised version of the manuscript. All authors read and approved the final manuscript.

## Availability of data and materials

The data set is available from Farah Naz Qamar.

## Consent for publication

All authors agreed to the publication of this work.

## Declaration of Competing Interest

All the authors declare that they have no conflict of interest, whether financial or otherwise.

## References

[1] Renzi S., Langenberg-Ververgaert K.P.S., Waespe N. (2020). Primary immunodeficiencies and their associated risk of malignancies in children: an overview. Eur J Pediatr.

[2] Raje N., Dinakar C. (2015). Overview of immunodeficiency disorders. Immunol Allergy Clin.

[3] Bousfiha A., Jeddane L., Picard C. (2018). The 2017 IUIS phenotypic classification for primary immunodeficiencies. J Clin Immunol.

[4] Modell V., Orange J.S., Quinn J., Modell F. (2018). Global report on primary immunodeficiencies: 2018 update from the Jeffrey Modell Centers Network on disease classification, regional trends, treatment modalities, and physician reported outcomes. Immunol Res.

[5] Al-Herz W. (2008). Primary immunodeficiency disorders in Kuwait: first report from Kuwait national primary immunodeficiency registry (2004-2006). J Clin Immunol.

[6] Aghamohammadi A., Mohammadinejad P., Abolhassani H. (2014). Primary immunodeficiency disorders in Iran: update and new insights from the third report of the national registry. J Clin Immunol.

[7] Al-Saud B., Al-Mousa H., Al Gazlan S. (2015). Primary immunodeficiency diseases in Saudi arabia: a tertiary care hospital experience over a period of three years (2010-2013). J Clin Immunol.

[8] El-Sayed Z.A., Abramova I., Aldave J.C. (2019). X-linked agammaglobulinemia (XLA):Phenotype, diagnosis, and therapeutic challenges around the world. World Allergy Organ J.

[9] Wallace J.G., Tipu H.N., Stafstrom K. (2019). Rethinking newborn screening for severe combined immunodeficiency: lessons from an international partnership for patients with primary immunodeficiencies in Pakistan. Clin Immunol.

[10] Qamar F., Junejo S., Qureshi S. (2017). A novel mutation in the JH4 domain of JAK3 causing severe combined immunodeficiency complicated by vertebral osteomyelitis. Clin Immunol.

[11] Qureshi S., Sheikh M.D.A., Qamar F.N. (2019). Autosomal Recessive Agammaglobulinemia - first case with a novel TCF3 mutation from Pakistan. Clin Immunol.

[12] Seleman M., Hoyos-Bachiloglu R., Geha R.S., Chou J. (2017). Uses of next-generation sequencing technologies for the diagnosis of primary immunodeficiencies. Front Immunol.

[13] de Silva N.R., Gunawardena S., Rathnayake D., Wickramasingha G.D. (2013). Spectrum of primary immunodeficiency disorders in Sri Lanka. Allergy Asthma Clin Immunol.

[14] Shah I. (2005). Hyper IgM syndrome with tuberculous osteomyelitis and scrofuloderma. Indian Pediatr.

[15] Modell V., Knaus M., Modell F., Roifman C., Orange J., Notarangelo L.D. (2014). Global overview of primary immunodeficiencies: a report from Jeffrey Modell Centers worldwide focused on diagnosis, treatment, and discovery. Immunol Res.

[16] Lederman H.M. (2000). The clinical presentation of primary immunodeficiency diseases. Clin Focus Prim Immunol Def.

[17] Azizi G., Ahmadi M., Abolhassani H. (2016). Autoimmunity in primary antibody deficiencies. Int Arch Allergy Immunol.

[18] Verma S., Sharma P.K., Sivanandan S. (2008). Spectrum of primary immune deficiency at a tertiary care hospital. Indian J Pediatr.

[19] Sobh A., Bonilla F.A. (2016). Vaccination in primary immunodeficiency disorders. J Allergy Clin Immunol Pract.

[20] Shahmohammadi S., Saffar M.J., Rezai M.S. (2014). BCG-osis after BCG vaccination in immunocompromised children: case series and review. J Pediat Rev.

[21] Ameratunga R., Woon S.T., Neas K., Love D.R. (2010). The clinical utility of molecular diagnostic testing for primary immune deficiency disorders: a case based review. Allergy Asthma Clin Immunol.

